# Effects of Blue and Green Light on Pigment Content, Photosynthesis, and Chloroplast Structure in *CRY1a*-Deficient Tomato Mutants

**DOI:** 10.3390/plants15213297

**Published:** 2026-10-26

**Authors:** Mikhail Vereshchagin, Diana Meister, Galina Glushko, Anna Abramova, Lyudmila Khalilova, Alexander Voronkov, Vladimir Kuznetsov, Vladimir Kreslavski, Anatoly Kosobryukhov, Pavel Pashkovskiy

**Affiliations:** 1 K.A. Timiryazev Institute of Plant Physiology, Russian Academy of Sciences, Moscow 127276, Russialhalilova@mail.ru (L.K.); voronkov_as@mail.ru (A.V.);; 2 Institute of Basic Biological Problems, Russian Academy of Sciences, Pushchino 142290, Russia

**Keywords:** A/Ci curves, chloroplasts, CRY1a, phenylpropanoid biosynthesis, photosynthesis rate, photosynthetic pigments, *Solanum lycopersicum*, stomatal conductance

## Abstract

Cryptochrome 1a (CRY1a) mediates blue-light signalling in tomato, but its contribution to acclimation to combined blue and green light remains unresolved. We compared wild-type (WT) and *cry1* plants under white fluorescent light (WFL), blue light (BL), green light (GL), and BL/GL using gas exchange, A/Ci curves, chlorophyll fluorescence, pigment composition, electron microscopy, transcript abundance, and phenolic metabolism. Under BL, *cry1* had a lower photosynthesis rate, stomatal conductance, and Y(II), despite higher chlorophyll content and grana area. Under BL/GL, the photosynthesis rate and stomatal conductance were approximately 35% lower in *cry1*, and Vcmax, Jmax, and TPU decreased by 48–57%. Y(II) was lower, and Y(NO) was higher in *cry1* than in WT. Under BL/GL, the grana area was lower in *cry1*, whereas the starch-grain area, stroma area, and plastoglobule number showed small differences that were not statistically significant. Transcript responses varied among spectra and functional groups. These results support a contribution of CRY1a to the coordination of structural and physiological acclimation to light quality. The BL/GL response is consistent with an imbalance between CO_2_ supply and carbon utilization that may promote photoprotective feedback.

## 1. Introduction

Light provides the energy required for photosynthesis and regulates leaf development and functional status. Blue light (BL) is absorbed predominantly in the upper leaf layers, whereas green light (GL) penetrates more deeply and can support photochemical reactions and contribute to the photosynthesis rate in the inner mesophyll layers [1,2]. At the same photon flux density, BL and GL differ in terms of the spatial distribution of excitation within the leaf and may elicit distinct signalling responses [3,4]. Cryptochromes perceive BL and near-ultraviolet radiation through a flavin chromophore. Four cryptochromes have been identified in tomato: CRY1a, CRY1b, CRY2, and CRY3. CRY1a is the principal CRY1-type cryptochrome in vegetative organs and contributes to photomorphogenesis, the regulation of pigment composition, and the tolerance of the photosynthetic apparatus to high irradiance [5,6]. CRY1a also regulates phytohormone homeostasis and carotenoid metabolism in tomato fruits [5]. CRY1b encodes a truncated copy of CRY1a that lacks most of the C-terminal extension; this gene arose through whole-genome triplication in the common ancestor of the Solanaceae [6]. Activated CRY1 interacts with the COP1/SPA complex and alters the stability and activity of transcriptional regulators, including HY5 and PIF4. The loss of CRY1a may therefore simultaneously affect photomorphogenesis, phenylpropanoid biosynthesis, development of the photosynthetic apparatus, and antioxidant defence [7,8,9,10].

GL is both a less strongly absorbed component of the photosynthetically active spectrum and a component of light signalling. Cryptochromes can sense changes in the BL-to-GL ratio, and GL can attenuate certain BL-induced responses [11,12]. BL-dependent stomatal opening is initiated primarily by phototropins; the signal is transmitted through BLUE LIGHT SIGNALING 1 (BLUS1) kinase to the plasma membrane H^+^-ATPase in guard cells. Cryptochromes also help maintain stomata in an open state during the light period [13,14,15]. GL can attenuate BL-induced stomatal opening, although the direction and magnitude of this effect depend on the spectral background and photon-flux ratio. Therefore, the presence of GL does not necessarily induce stomatal closure.

In tomato and other C3 plants, the spectral composition of light affects pigment content, chloroplast organization, gas exchange, and stomatal function. Increasing the proportion of BL generally enhances stomatal opening and may support a higher photosynthesis rate, whereas replacing some BL with GL often attenuates or alters these effects depending on the contributions of cryptochromes and phototropins and on the mesophyll status [4,14,15,16]. In comparable experimental systems, changes in the maximum Rubisco carboxylation rate, maximum electron transport rate, and triose phosphate utilization rate have been attributed to the combined effects of limitations on carboxylation, electron transport, and carbon allocation rather than to an isolated change in a single stage of photosynthesis [17,18,19]. It remains unclear how CRY1a-associated differences in light harvesting and protective metabolism relate to stomatal and mesophyll responses during acclimation to these spectra.

Previous studies have shown that tomato CRY1a contributes to the maintenance of photosynthesis and antioxidant defence under high-intensity BL and UV-B, but the responses to moderate BL, GL, and their combined action have received less attention [20,21,22]. Phenolic metabolites can contribute to UV screening and antioxidant defence, and earlier work in tomato photoreceptor mutants linked these protective responses to light signalling [20,21]. We therefore measured UV-absorbing compounds, total phenolic and flavonoid contents, and ABTS/DPPH antiradical activity to examine whether photosynthetic acclimation was accompanied by changes in putative protective metabolites and extract radical-scavenging capacity. These assays address complementary chemical properties; they do not directly measure in vivo reactive oxygen species or establish UV protection under the present growth conditions.

The working hypothesis was that the *cry1* line would differ from WT in the coordination of stomatal and mesophyll responses during acclimation to BL and GL. We predicted that BL/GL acclimation could reveal concurrent differences in stomatal conductance, photosynthetic parameters, and photochemical energy use. The aim was to compare the physiological responses of WT and the CRY1a-deficient line to WFL, BL, BL/GL, and GL. We examined whether changes in photosynthesis rate (Pn) were associated primarily with stomatal conductance or were accompanied by changes in parameters calculated from A/Ci curves and photochemical activity. We also examined pigment content; chloroplast ultrastructure; transcript levels of genes involved in light signalling, phenylpropanoid biosynthesis, photoprotection, and stomatal regulation; and phenolic compound content and antiradical activity in conjunction with these functional responses.

## 2. Results

**Figure 1:**
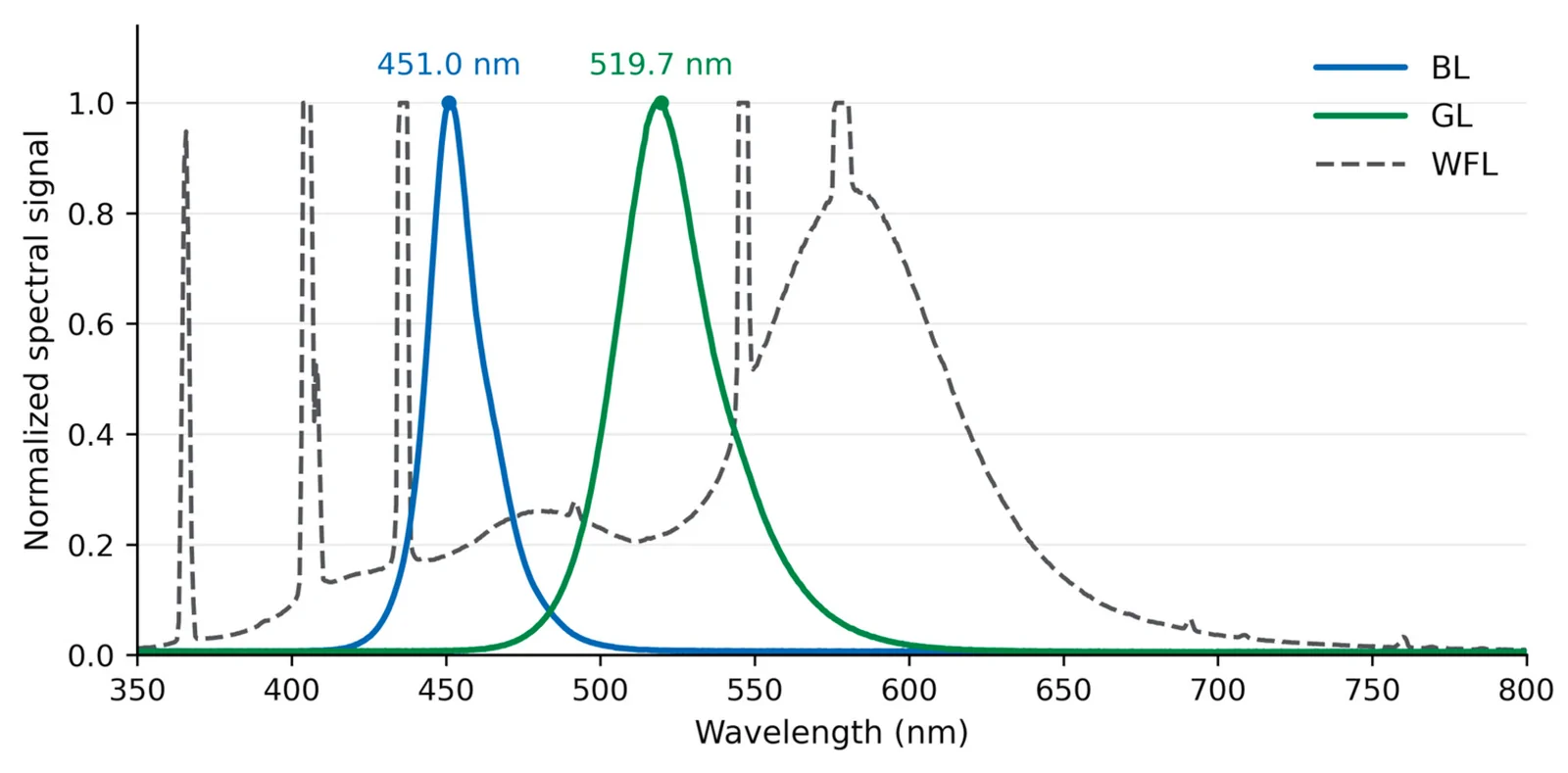
Emission spectra of the blue (BL) and green (GL) LEDs and white fluorescent lamps (WFL). Each spectral signal was normalized to its maximum within 350–800 nm. Curves were plotted without smoothing. BL and GL peaks were 451.0 and 519.7 nm, with full widths at half maximum of 20.0 and 35.9 nm, respectively. The BL/GL treatment used the BL and GL sources at a 1:1 photon-flux ratio (Section 4.1).

The emission spectra of the light sources are shown in Figure 1. BL and GL had emission maxima at 451.0 and 519.7 nm, respectively, whereas WFL covered a broader wavelength range.

### 2.1. Gas Exchange and Photosynthetic Parameters

**Figure 2:**
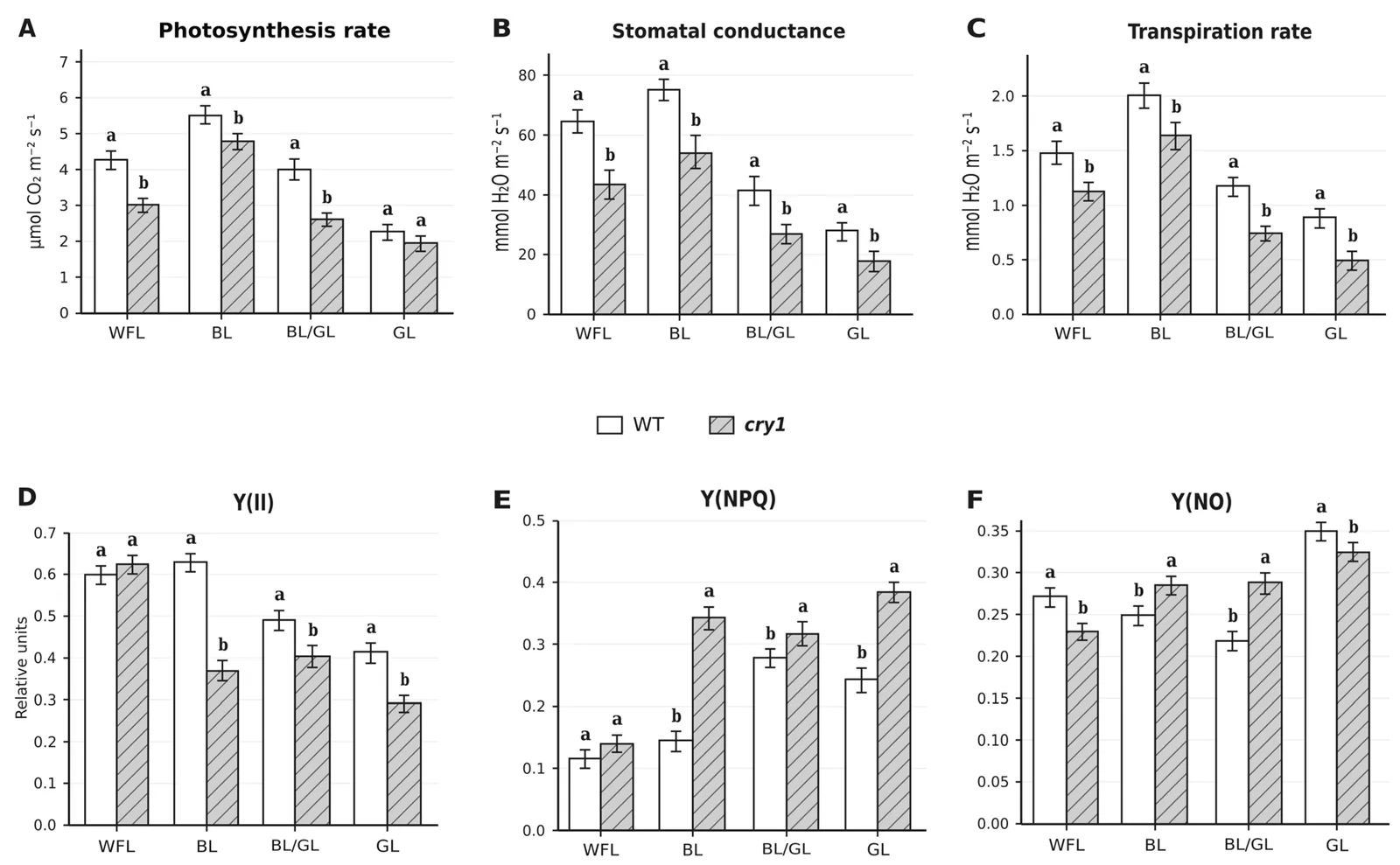
Gas exchange and chlorophyll a fluorescence variables in WT and *cry1* plants under WFL, BL, BL/GL, and GL: (**A**) photosynthesis rate; (**B**) stomatal conductance; (**C**) transpiration rate; (**D**) Y(II); (**E**) Y(NPQ); (**F**) Y(NO). Bars show means ± SE of four independent biological replicates (*n* = 4). Genotypes were compared separately within each light treatment using one-way ANOVA followed by Duncan’s multiple range test (*p* < 0.05). Different letters indicate significant differences; identical letters indicate nonsignificant differences. For panels (**D**–**F**), all leaves were measured under the same blue actinic light (450 nm; 250 µmol photons m^−2^ s^−1^), after 30 min of dark adaptation and 10 min of actinic illumination.

The gas exchange variables differed among the genotype and growth-light treatment combinations (Figure 2A–C). In WT, the highest photosynthesis rate, stomatal conductance, and transpiration rate occurred under BL. In *cry1*, these variables were lower than in WT under WFL, BL, and BL/GL. The greatest relative reduction in photosynthesis rate occurred under BL/GL, where the value in *cry1* was approximately 35% lower than in WT. The reductions in stomatal conductance and transpiration rate under this treatment indicate a difference in the stomatal response of *cry1*.

Stomatal conductance and transpiration rates were lower in *cry1* than in WT under WFL, BL, BL/GL, and GL (Figure 2B,C). Under BL/GL, stomatal conductance decreased by approximately 35%, and the transpiration rate decreased by approximately 40%. Photosynthesis rate and stomatal conductance showed similar relative decreases under this treatment; the relative stomatal and non-stomatal contributions to the decrease in photosynthesis rate cannot be resolved from these comparisons alone.

Under WFL and BL, the intergenotypic differences in the estimated maximum Rubisco carboxylation rate (Vcmax), electron transport parameter (Jmax), and triose phosphate utilization rate (TPU) were not statistically significant (Table 1). Under BL/GL, these values were 48–57% lower in *cry1* than in WT, and carboxylation efficiency (CE) decreased by 63.5%, from 0.085 to 0.031 µmol CO_2_ m^−2^ s^−1^ Pa^−1^. Under GL, the intergenotypic differences in Vcmax, Jmax, and TPU were not statistically significant. Together with the decrease in stomatal conductance, the findings under BL/GL indicate concurrent changes in stomatal CO_2_ influx and photosynthetic parameters.

### 2.2. Chlorophyll a Fluorescence

Chlorophyll a fluorescence parameters were measured under a common blue actinic light (450 nm; 250 µmol photons m^−2^ s^−1^) after acclimation to the four growth-light treatments (Figure 2D–F). Under WFL, Y(II) and Y(NPQ) did not differ significantly between WT and *cry1*, whereas Y(NO) was lower in *cry1*. Under BL and BL/GL, Y(II) was lower in *cry1*, whereas Y(NPQ) and Y(NO) were higher. Under GL, the decrease in Y(II) was accompanied by higher Y(NPQ) and lower Y(NO). The significance of the genotype comparisons is indicated by the letters in Figure 2D–F.

### 2.3. Photosynthetic Pigments and Chloroplast Ultrastructure

#### 2.3.1. Photosynthetic Pigments

**Figure 3:**
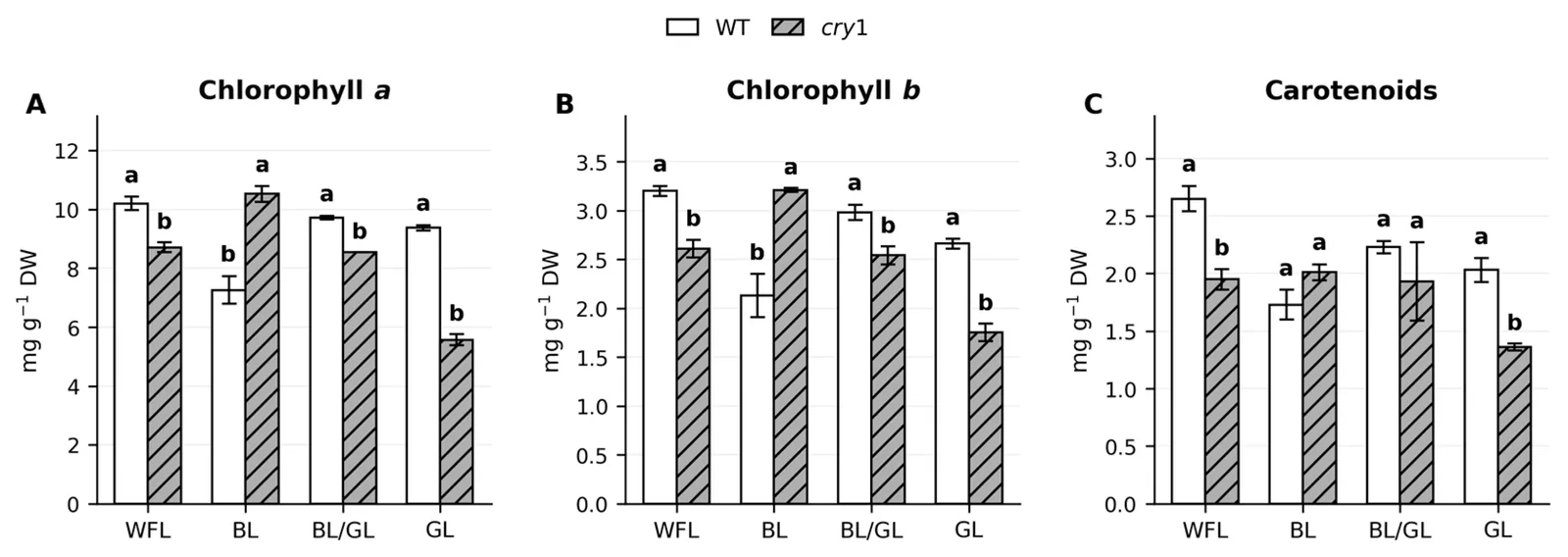
Photosynthetic pigment contents in leaves of WT and *cry1* plants under WFL, BL, BL/GL, and GL: (**A**) chlorophyll a; (**B**) chlorophyll b; (**C**) total carotenoids. Bars show means ± SE of four independent biological replicates (*n* = 4). Within each light treatment, different letters indicate significant differences between genotypes according to one-way ANOVA followed by Duncan’s multiple range test (*p* < 0.05); identical letters indicate nonsignificant differences.

Under WFL, the chlorophyll a content in *cry1* was 8.71 mg g^−1^ dry weight compared with 10.20 mg g^−1^ in WT, corresponding to a 14.6% decrease. The chlorophyll b contents were 2.61 and 3.20 mg g^−1^, respectively, and the carotenoid contents were 1.95 and 2.65 mg g^−1^; the decreases in the mutant were 18.4% and 26.4%, respectively. All the differences were statistically significant (Figure 3A–C). Under BL, the chlorophyll a and b contents in *cry1* increased to 10.52 and 3.21 mg g^−1^, respectively, corresponding to increases of 44.9% and 50.7% relative to WT. The carotenoid content was 16.2% greater in the mutant than in the WT at 2.01 mg g^−1^ and 1.73 mg g^−1^, respectively, although the difference was not statistically significant (Figure 3A–C).

Under BL/GL, the chlorophyll a content in *cry1* decreased by 12.1% to 8.54 mg g^−1^ compared with 9.72 mg g^−1^ in WT. The chlorophyll b content decreased by 14.8% to 2.54 mg g^−1^ compared with 2.98 mg g^−1^; both differences were statistically significant. The carotenoid content decreased by 13.5% to 1.93 mg g^−1^ compared with 2.23 mg g^−1^, but the difference was not statistically significant (Figure 3A–C). Under GL, the contents of all three pigment groups were lower in *cry1* than in WT. Chlorophyll a contents were 5.57 and 9.37 mg g^−1^; chlorophyll b contents were 1.753 and 2.66 mg g^−1^; and carotenoid contents were 1.36 and 2.03 mg g^−1^. The corresponding decreases in the mutant were 40.6%, 34.1%, and 33.0%, respectively. All the differences were statistically significant (Figure 3A–C). Thus, a statistically significant increase in the chlorophyll content in *cry1* was observed only under BL, whereas the carotenoid content did not increase significantly under any light treatment.

#### 2.3.2. Chloroplast Ultrastructure

**Figure 4:**
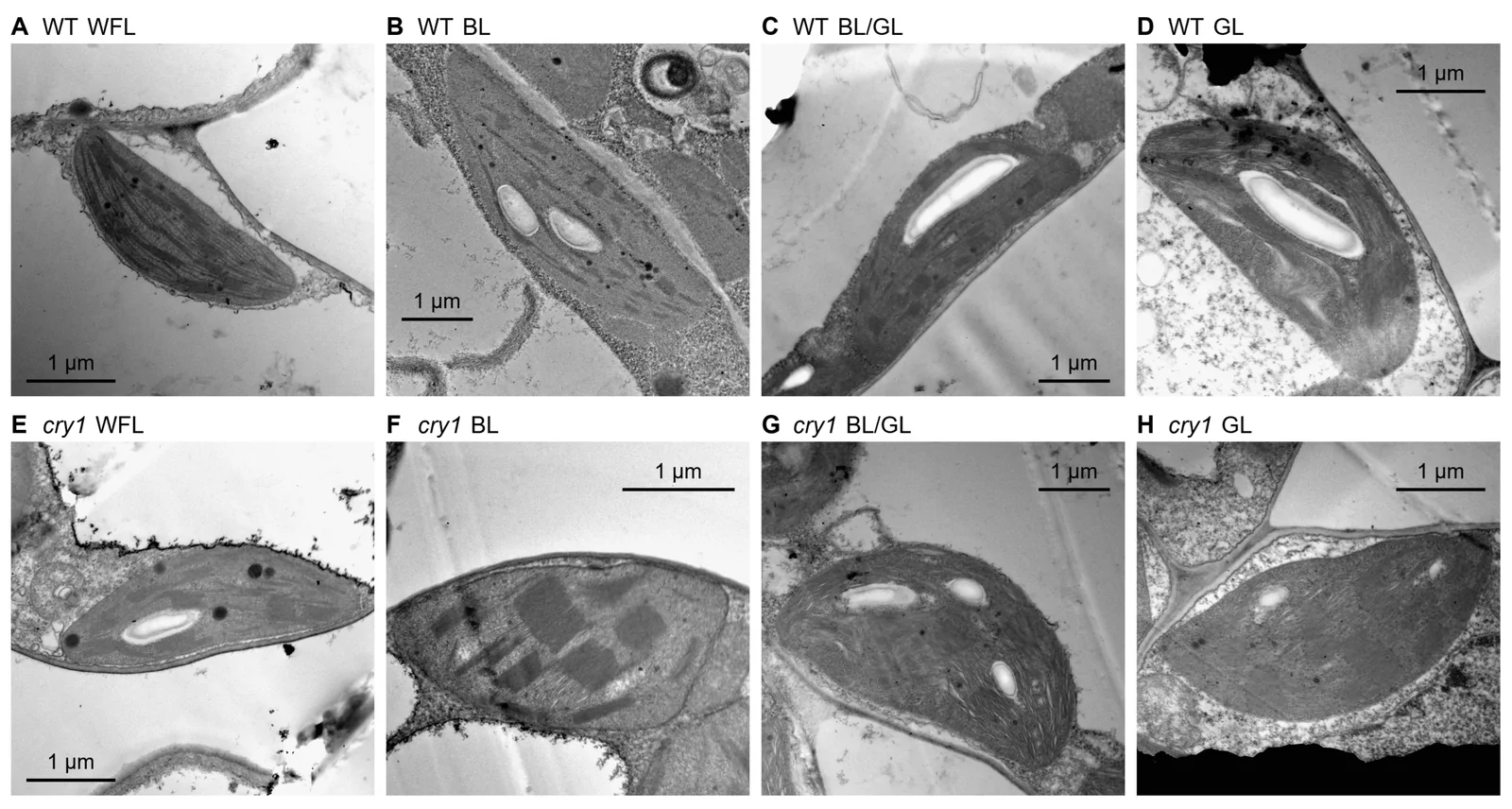
Chloroplast ultrastructure under the four growth-light treatments: (**A**–**D**) WT and (**E**–**H**) *cry1* under WFL, BL, BL/GL, and GL, respectively, from left to right. Scale bars, 1 µm. Morphometry for all four growth-light treatments is reported in Figure 5A–D.

**Figure 5:**
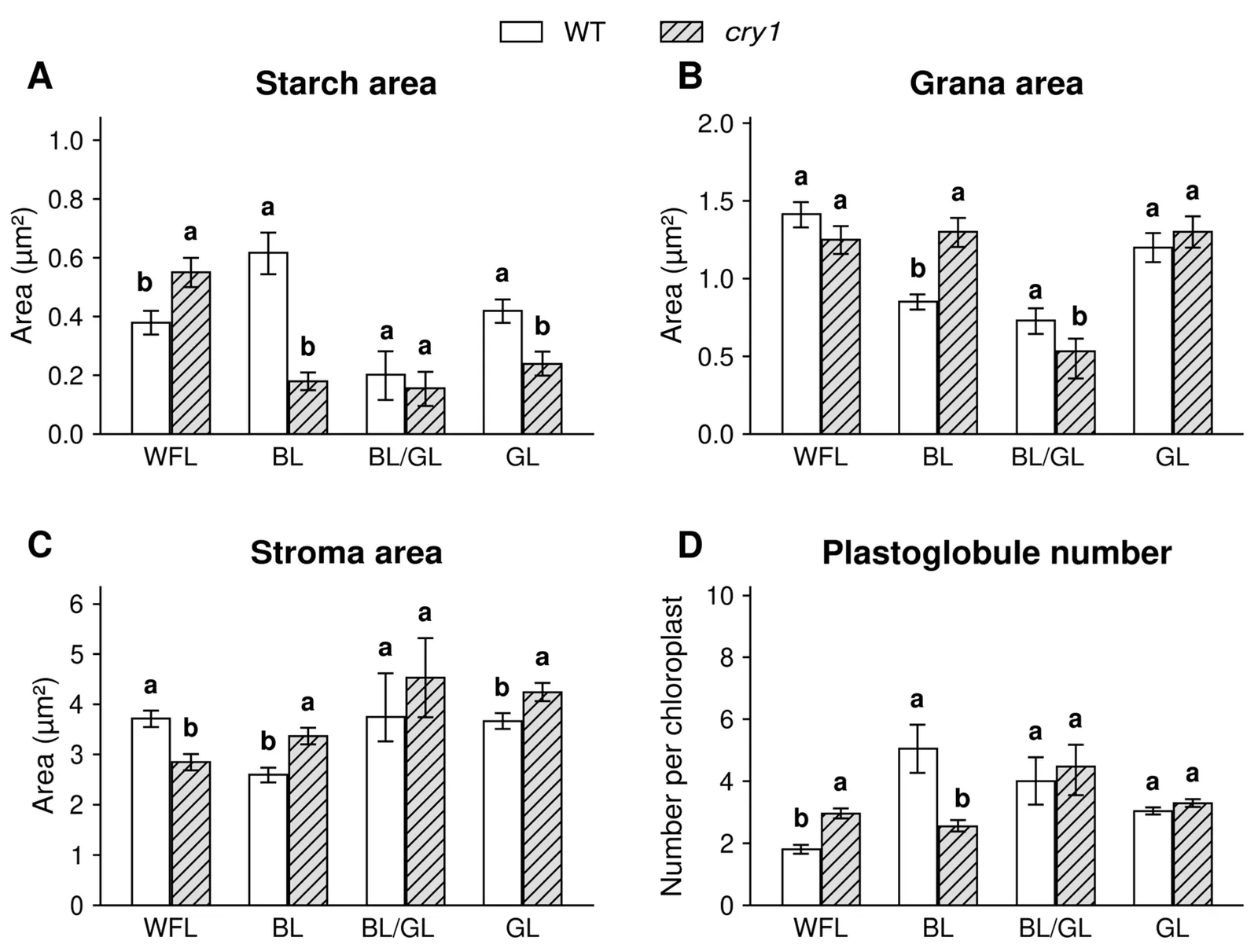
Chloroplast morphometry in WT and *cry1* under WFL, BL, BL/GL, and GL: (**A**) starch-grain area; (**B**) grana area; (**C**) stroma area; (**D**) plastoglobule number. Bars show means ± SE of four independent biological replicates (*n* = 4) for each genotype-by-light-treatment combination. Five sections and 20 micrographs were analysed for each experimental point. Within each light treatment, genotypes were compared using one-way ANOVA followed by Duncan’s multiple range test (*p* < 0.05). Different letters indicate significant differences; identical letters indicate nonsignificant differences. Representative BL/GL micrographs are shown in Figure 4C,G.

Representative transmission electron micrographs show WT and *cry1* under WFL (Figure 4A,E), BL (Figure 4B,F), BL/GL (Figure 4C,G), and GL (Figure 4D,H). Morphometry for all four growth-light treatments is presented in Figure 5A–D.

Under WFL, the stroma area in the mutant decreased from 3.71 to 2.85 µm^2^, whereas the number of plastoglobules increased (Figure 4A,E and Figure 5C,D). The starch-grain area was also significantly higher in *cry1*, whereas the grana area did not differ significantly (Figure 5A,B).

Under BL, the starch-grain area and plastoglobule number were lower in *cry1*, whereas the grana area was greater. Stroma area increased from 2.60 to 3.36 µm^2^ (Figure 4B,F and Figure 5A–D).

Under GL, the starch-grain area in *cry1* was 43% lower than in WT, whereas the stroma area was significantly greater in *cry1* (Figure 5A,C). The grana area and plastoglobule number did not differ significantly between genotypes (Figure 4D,H and Figure 5B,D).

Under BL/GL, the grana area was lower in *cry1* than in WT (Figure 5B). The starch-grain area, stroma area, and plastoglobule number showed small differences that were not statistically significant (Figure 5A,C,D). Representative micrographs are shown in Figure 4C,G.

### 2.4. Relative Transcript Levels

**Figure 6:**
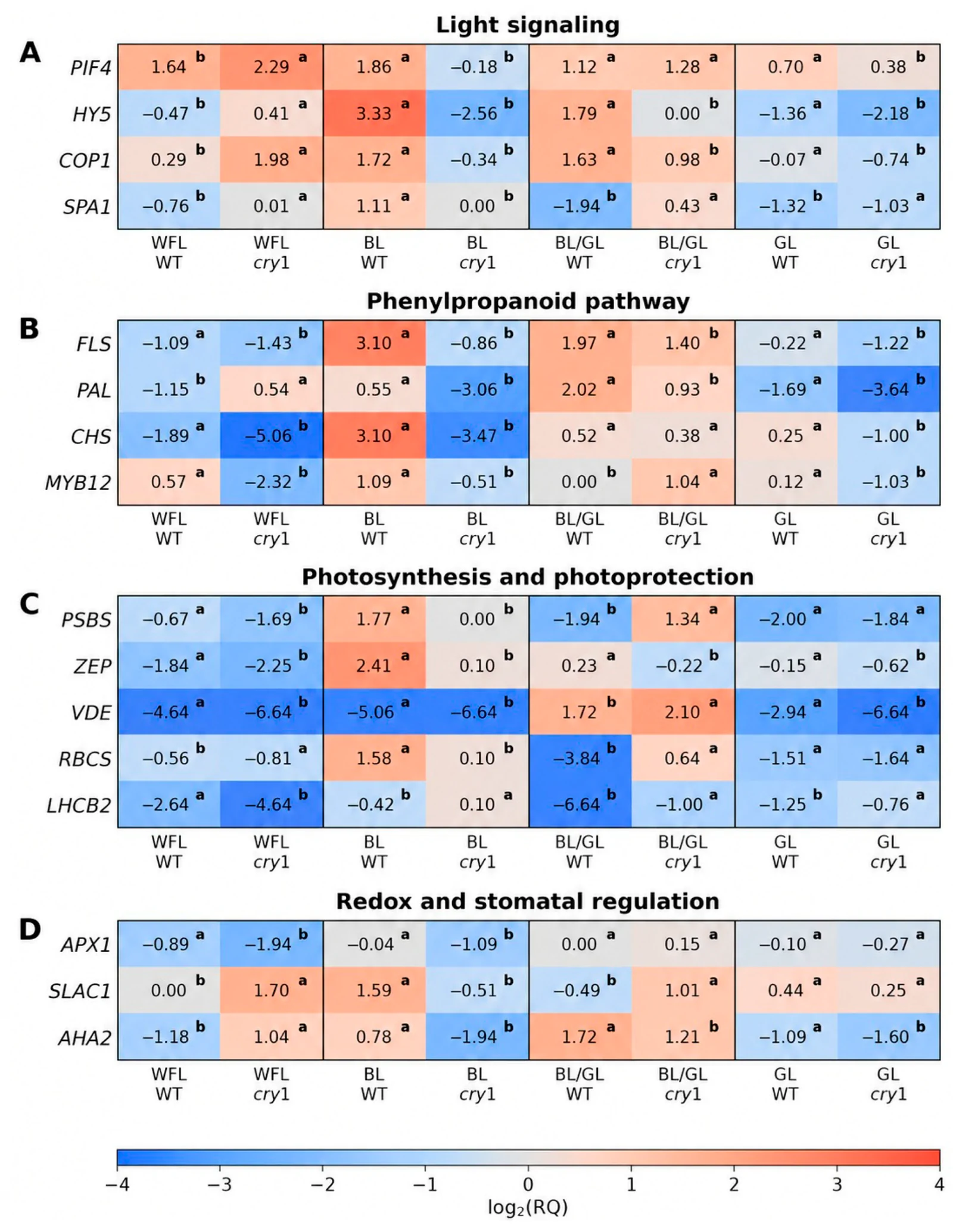
Relative transcript levels in WT and *cry1* plants under WFL, BL, BL/GL, and GL: (**A**) light-signalling regulators; (**B**) phenylpropanoid-biosynthesis genes; (**C**) photosynthesis and photoprotection; (**D**) antioxidant defence and stomatal regulation. Colours and numbers indicate mean log_2_(RQ) values; the colour scale is limited to −4 to +4. RT–qPCR was performed using four independent biological replicates (*n* = 4), each with at least three technical reactions. Within each light treatment, genotypes were compared using one-way ANOVA followed by Duncan’s multiple range test (*p* < 0.05). Different letters indicate significant differences. Means ± SE are presented in Table S2.

The observed differences in relative transcript levels between genotypes varied among growth-light treatments (Figure 6; Table S2). Under WFL, the transcript levels of *PIF4, HY5, COP1, SPA1, SLAC1*, and *AHA2* were greater in *cry1*. Concurrently, the transcript levels decreased for most of the phenylpropanoid biosynthesis genes examined, all the measured photosynthesis and photoprotection genes, and *APX1*.

Under BL, within-treatment comparisons indicated intergenotypic differences for all measured transcripts. In *cry1*, transcript levels decreased for the light-signalling regulators *PIF4, HY5, COP1*, and *SPA1*; the phenylpropanoid-biosynthesis genes *PAL, CHS, FLS*, and *MYB12*; the photoprotection genes *PSBS, ZEP*, and *VDE*; and *RBCS, APX1, SLAC1*, and *AHA2*. *LHCB2* was the exception, with a 1.43-fold higher transcript level in *cry1*.

Under BL/GL, the transcript levels of *PSBS, VDE, RBCS*, and *LHCB2* increased in *cry1,* whereas those of *ZEP* decreased. Among the phenylpropanoid-biosynthesis genes, *MYB12* increased, *PAL* and *FLS* decreased, and *CHS* did not differ between genotypes. *AHA2* decreased, whereas *SLAC1* increased. Under GL, the transcript levels of most genes decreased in *cry1*; *LHCB2* and *SPA1* increased, while those of *PSBS, RBCS, APX1*, and *SLAC1* did not differ significantly between genotypes.

### 2.5. Phenolic Compounds and Antiradical Activity

**Figure 7:**
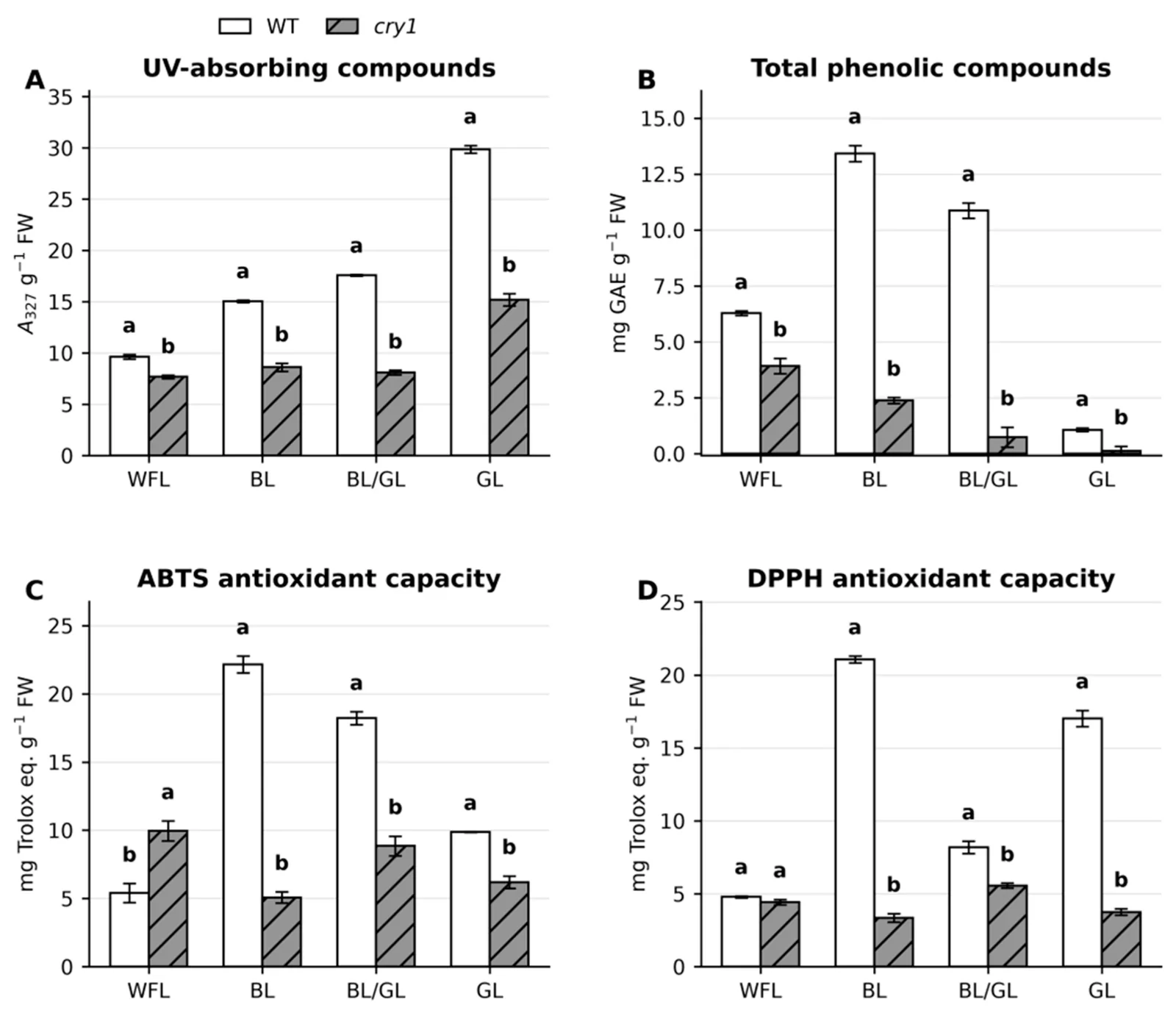
Phenolic compound contents and antiradical activity in leaves of WT and *cry1* plants under WFL, BL, BL/GL, and GL: (**A**) UV-absorbing compounds; (**B**) total phenolic compounds; (**C**) activity in the ABTS assay; (**D**) activity in the DPPH assay. Bars show means ± SE of four independent biological replicates (*n* = 4). Within each light treatment, different letters indicate significant differences between genotypes according to one-way ANOVA followed by Duncan’s multiple range test (*p* < 0.05); identical letters indicate nonsignificant differences.

The content of UV-absorbing compounds in *cry1* was 20% lower than that in WT under WFL, 43% lower under BL, 54% lower under BL/GL, and 49% lower under GL (Figure 7A). Under GL, the value was 29.87 A_327_ g^−1^ fresh weight in WT and 15.19 in *cry1*. Absorbance at 327 nm is used here as an operational index of extractable UV-absorbing compounds, rather than a direct measure of leaf UV-screening efficiency.

The total phenolic content in *cry1* was 38% lower than that in WT under WFL, 82% lower under BL, 93% lower under BL/GL, and 88% lower under GL (Figure 7B). Under BL, the respective values were 13.43 and 2.38 mg gallic acid equivalents per gram fresh weight.

The total flavonoid content under WFL was 0.98 arbitrary units in WT and 1.15 in *cry1*; this difference was not statistically significant. Under BL, the total flavonoid content increased from 1.99 in WT to 2.83 in *cry1*. Under GL, the respective values were 0.61 and 0.59, with no statistically significant difference between genotypes.

In the ABTS assay, antiradical activity in *cry1* was 1.85-fold higher under WFL but 77%, 51%, and 37% lower than in WT under BL, BL/GL, and GL, respectively (Figure 7C). In the DPPH assay, the genotypes did not differ under WFL, whereas *cry1* activity was 84%, 32%, and 78% lower under BL, BL/GL, and GL, respectively (Figure 7D).

## 3. Discussion

The WT–*cry1* comparison revealed concurrent differences in gas exchange, photosystem II energy partitioning, pigment accumulation, chloroplast ultrastructure, transcript levels, and phenolic metabolism across the tested growth spectra. These results support a contribution of CRY1a to spectral acclimation and are consistent with evidence that cryptochromes regulate several components of the light response in tomato and other higher plants [6,9,23].

These gas exchange data are consistent with a role for CRY1a in BL-dependent stomatal regulation. Under BL, WT had a higher photosynthesis rate, stomatal conductance, and transpiration rate than *cry1* (Figure 2A–C). These findings are consistent with BL-induced stomatal opening through phototropins, BLUS1, and the plasma-membrane H^+^-ATPase and with the contribution of cryptochromes to maintaining this response during the light period [4,13,15].

The greatest relative reduction in photosynthesis rate in *cry1* compared with WT occurred under BL/GL. Gas exchange rates were lower under BL/GL than under BL in both genotypes; under GL, the photosynthesis rate was lower still (Figure 2A–C). Cryptochromes sense the BL-to-GL ratio, and GL affects BL-dependent growth and photomorphogenic responses [11,12,16]. The present gas exchange pattern is consistent with such spectral modulation.

CRY1 contributes to acclimation of the photosynthetic apparatus to high irradiance [24], and CRY1-dependent light responses are modulated by the BL-to-GL ratio [11]. In Arabidopsis thaliana, Kreslavski et al. [25] found that CRY1 deficiency lowered the photosynthesis rate under a red/blue spectrum, whereas supplemental GL reduced or eliminated the difference between the mutant and WT. A decrease in CRY1 activity under GL may therefore contribute to the smaller, nonsignificant genotype difference in photosynthesis rate observed under GL in the present study. Under BL/GL, lower stomatal conductance and lower Vcmax, Jmax, and TPU in *cry1* coincided with the greatest relative reduction in photosynthesis rate. We propose that the response to combined BL and GL involves altered coordination of CO_2_ supply and carbon utilization, leading to the pronounced physiological differences between *cry1* and WT under this treatment.

The parameters calculated from A/Ci curves further clarify the photosynthetic response. Intergenotypic differences in Vcmax, Jmax, and TPU were not statistically significant under WFL or BL, whereas these parameters and CE were lower in *cry1* under BL/GL (Table 1). Under GL, Vcmax, Jmax, and TPU did not differ significantly between genotypes. These photosynthetic parameters were estimated from CO_2_-response curves measured at constant irradiance. Because the calculations used intercellular rather than chloroplast CO_2_ concentrations, the estimates may also reflect variation in mesophyll conductance.

Under BL/GL, Vcmax, Jmax, and TPU in *cry1* were 47.5%, 51.9%, and 56.7% lower than the respective WT values, and CE was 63.5% lower (Table 1). Similar relative changes in Vcmax and Jmax maintained the Jmax/Vcmax ratio within a comparable range: 3.24 in WT and 2.97 in *cry1*. This ratio reflects the balance between the potential electron transport rate supporting RuBP regeneration and the maximum carboxylation rate [26]. The BL/GL results therefore indicate simultaneous decreases in several photosynthetic parameters.

Under GL, the estimated Vcmax and Jmax were lower than those under BL for both genotypes. On the basis of the mean values, Vcmax decreased by 32.6% in WT and 36.7% in *cry1*, whereas Jmax decreased by 50.9% and 54.5%, respectively. These differences were observed alongside lower gas exchange rates under GL than under BL, but the cause of the spectral response was not identified. The reduction in TPU in *cry1* under BL/GL is consistent with the restricted downstream utilization of carbon-fixation products [19].

Fluorescence parameters characterize photochemical energy use and energy partitioning in photosystem II after acclimation to the growth-light treatments. All treatments were measured using the same blue actinic light. Under WFL, Y(II) and Y(NPQ) did not differ significantly between genotypes, whereas Y(NO) was lower in *cry1*. Under BL and BL/GL, lower Y(II) in *cry1* coincided with higher Y(NPQ) and Y(NO) (Figure 2D–F). Under BL/GL, lower Y(II) also coincided with lower stomatal conductance and lower photosynthetic parameters [18,27]. Under GL, lower Y(II) was accompanied by higher Y(NPQ) and lower Y(NO), indicating redistribution of excitation energy toward regulated dissipation.

Pigment composition differed between the lines across growth-light treatments. Under BL, the chlorophyll content per unit dry weight increased in *cry1*, whereas photosynthesis rate and Y(II) decreased (Figure 2A,D; Figure 3A,B). Thus, increased light-harvesting pigment content was not accompanied by an increased photosynthesis rate.

Ultrastructural observations provide further context for these functional responses. Under BL, the grana area increased in *cry1*, whereas the starch-grain area decreased (Figure 4B,F and Figure 5A,B). The grana area reflects the organization of the thylakoid system and light-harvesting apparatus, whereas the starch-grain area reflects the balance between the synthesis of photosynthetic products, their export, and their subsequent use in respiration and growth [4,19]. These contrasting changes describe distinct aspects of chloroplast acclimation.

Comparisons of pigment composition, ultrastructure, and functional variables revealed a mismatch between the structural and functional acclimation of the *cry1* photosynthetic apparatus to BL. The increases in chlorophyll a and b contents and the grana area were accompanied by decreases in the photosynthesis rate and Y(II), whereas no statistically significant increase in the total carotenoid content was detected (Figure 2A,D, Figure 3A–C and Figure 5B). Thus, chlorophyll accumulation and greater grana area were not accompanied by improved efficiency of photochemical energy conversion or a higher photosynthesis rate. The increase in Y(NPQ) under BL shows that a larger proportion of the excitation energy was dissipated through regulated pathways (Figure 2E).

The BL/GL micrographs extend the structural comparison to the treatment with the greatest relative reduction in photosynthesis rate in *cry1*. Representative WT and cry1 micrographs are shown in Figure 4C and Figure 4G, respectively, and quantitative comparisons are presented in Figure 5A–D. In contrast to the increase under BL, the grana area was lower in *cry1* under BL/GL (Figure 5B). This structural difference coincided with a lower photosynthesis rate and lower Y(II).

The mean starch-grain area under BL/GL was lower in *cry1*, consistent in direction with its lower photosynthesis rate, although the difference in starch-grain area was not statistically significant (Figure 5A and Figure 2A). Starch in a fixed section reflects the balance among synthesis, degradation, and export, whereas gas exchange measures an instantaneous flux [19]. Thus, the starch measurement and photosynthesis rate describe related processes over different timescales.

Under BL/GL, the mean number of plastoglobules per chloroplast was slightly higher in *cry1*, but the genotype difference was not statistically significant (Figure 5D; representative micrographs, Figure 4C,G). Electron tomography has demonstrated structural coupling of plastoglobules to thylakoid membranes and localization of tocopherol cyclase within these particles [28]. Quantitative proteomics in Arabidopsis thaliana links plastoglobules with prenyl-lipid metabolism and redox-related functions [29]. These functions provide a physiological context for plastoglobule responses to light. Overall, BL/GL morphometry showed lower grana area in *cry1*, whereas starch-grain area, stroma area, and plastoglobule number showed small differences that were not statistically significant (Figure 5A–D).

CO_2_ limitation can promote over-reduction of components of the photosynthetic electron transport chain and activate nonphotochemical quenching (NPQ). Reduced carbon metabolism can decrease ATP and NADPH consumption, while feedback regulation of chloroplast ATP synthase promotes lumen acidification and energy-dependent thermal dissipation [30]. Under BL/GL, *cry1* showed lower stomatal conductance, photosynthesis rate, and Y(II), together with higher Y(NPQ) and Y(NO), than WT (Figure 2). This pattern is consistent with restricted CO_2_ supply and carbon utilization increasing excitation pressure and promoting photoprotective energy dissipation. We therefore propose that increased NPQ forms part of the protective feedback accompanying the lower photosynthesis rate under BL/GL.

Transcript-level responses also depended on the light spectrum. Under BL, the decrease in Y(II) in *cry1* occurred alongside reduced transcript levels of the photoprotection genes encoding photosystem II subunit S (*PSBS*), zeaxanthin epoxidase (*ZEP*), and violaxanthin de-epoxidase (*VDE*). Under BL/GL, *PSBS* and *VDE* transcript levels increased despite the decrease in Y(II) (Figure 2D,E; Figure 6C). Therefore, transcript abundance alone does not provide a sufficient explanation for the observed photoprotective responses [21,22,27].

Under BL/GL, higher *SLAC1* and lower *AHA2* transcript levels in *cry1* coincided with lower stomatal conductance. The SLAC1 anion channel contributes to stomatal closure, whereas the plasma-membrane H^+^-ATPase AHA2 promotes light-dependent stomatal opening [13,31,32]. The transcript pattern under BL/GL is therefore consistent with reduced stomatal opening. Under BL, both transcript levels decreased in *cry1*, indicating that their relationship with stomatal conductance depends on the light treatment.

Phenolic compound content and antiradical activity also differed between genotypes in a spectrum-dependent manner (Figure 7). Changes in *CHS, FLS, PAL*, and *MYB12* transcript levels occurred alongside changes in phenolic compound content and antiradical activity (Figure 6B; Figure 7A–D). These parallel responses are consistent with the reported roles of HY5/COP1 modules and cryptochromes in regulating the phenylpropanoid pathway and antioxidant defence [8,33,34].

The greatest relative reduction in photosynthesis rate and the pronounced differences in photosynthetic parameters under BL/GL are consistent with concurrent stomatal and metabolic limitations. In *cry1*, stomatal conductance, Vcmax, Jmax, TPU, and Y(II) decreased, whereas Y(NO) increased. Intergenotypic differences in Vcmax, Jmax, and TPU were not statistically significant under WFL, BL, or GL. Under BL/GL, the combination of lower stomatal conductance, lower photosynthetic parameters, and lower Y(II) accompanied the approximately 35% reduction in photosynthesis rate. We propose that spectral modulation of CRY1-dependent responses alters the balance between CO_2_ supply and carbon utilization, thereby contributing to the feedback described above.

A recent study in sorrel (*Rumex acetosa* L.) combined structural traits, pigment-related optical indices, and chlorophyll fluorescence measurements across contrasting LED spectra [35]. Its treatment-dependent responses provide a useful comparison for interpreting structural and functional acclimation together.

## 4. Materials and Methods

### 4.1. Plant Material and Light Treatments

*Solanum lycopersicum* L. cv. Moneymaker plants were used: the wild-type line LA2706 (WT) and the radiation-induced CRY1a loss-of-function line LA4359, obtained from the Tomato Genetics Resource Center (University of California, Davis, CA, USA). Molecular and photomorphogenic characterization of tomato *cry1* mutants was reported by Weller et al. [36], and LA4359 was characterized physiologically by Liu et al. [5]. The mutant line is hereafter designated *cry1*. Stock plants were grown for 45 days in perlite under white fluorescent light (WFL; MASTER TL-D Super 80 36W/840 lamps, Philips, Poland, Piła,) at a photosynthetic photon flux density (PPFD) of 300 µmol m^−2^ s^−1^, a 16 h light/8 h dark photoperiod, and 22 °C. Plants were irrigated with half-strength Hoagland–Arnon nutrient solution. Cuttings were rooted under WFL for 15 days and then transferred for 14 days to WFL, BL, BL/GL, or GL. BL and GL were supplied by LEDs (Epistar, Taiwan, China). The spectral characteristics of the light sources were determined using an AvaSpec-ULS2048CL-EVO spectrometer (Avantes, The Netherlands, Apeldoorn). The BL emission peak was 451.0 nm, with half-maximum boundaries at 443.0 and 463.0 nm (full width at half maximum, FWHM, 20.0 nm). The GL emission peak was 519.7 nm, with half-maximum boundaries at 502.8 and 538.7 nm (FWHM, 35.9 nm) (Figure 1). The boundary wavelengths were obtained by linear interpolation between the adjacent measured points at 50% of the recorded peak signal, without additional baseline subtraction; FWHM was calculated as the difference between the upper and lower boundaries. For plotting, each spectral signal was normalized to its maximum within 350–800 nm. During the spectral treatments, PPFD was approximately 400 µmol m^−2^ s^−1^ at the upper canopy and 300 µmol m^−2^ s^−1^ at the second and third leaf positions used for measurements. In BL/GL, the blue and green components each supplied 150 µmol m^−2^ s^−1^ at these leaf positions, giving a BL:GL photon-flux ratio of 1:1. The photoperiod was 16 h light/8 h dark, and the temperature was 22 °C in all treatments. Fully expanded leaves from the second and third nodes that developed during spectral treatment were analysed.

### 4.2. Gas Exchange and Photosynthetic Parameters

Photosynthesis rate (Pn), stomatal conductance (gs), and transpiration rate (E) were measured with a portable CIRAS-3 infrared gas analyser (PP Systems, USA, Amesbury) equipped with a 2.5 cm^2^ leaf chamber. Gas exchange at an ambient CO_2_ concentration of 400 µmol mol^−1^ was recorded at a photosynthetic photon flux density of 250 µmol m^−2^ s^−1^. A/Ci curves were obtained at the same irradiance: the ambient CO_2_ concentration was sequentially decreased from 400 to 200, 100, and 50 µmol mol^−1^; after a second measurement at 400 µmol mol^−1^, it was increased to 800, 1200, 1600, and 2000 µmol mol^−1^. Intercellular CO_2_ concentration was calculated by the instrument software from CO_2_ and water-vapour flux balances. Measurements at 400 µmol mol^−1^ CO_2_ and the A/Ci curves were analysed separately. Pn–Ci pairs were used to construct the curves; the repeated 400 µmol mol^−1^ point was excluded from the calculation dataset. 

Parameters were calculated from A/Ci curves using the C_3_ photosynthesis model, in which the photosynthesis rate is limited by Rubisco carboxylation, ribulose-1,5-bisphosphate regeneration, or triose phosphate utilization [17,18,19]:

$$A\;=\;\min\left(W_{c},W_{j},W_{p}\right)\;-\;R_{d},$$

$$W_{c}=\frac{V_{c\max}\left(C_{i}\;-\;\Gamma^{*}\right)}{C_{i}+K_{c}\left(1+O/K_{o}\right)},$$

$$W_{j\;}=\frac{J\left(C_{i}\;-\;\Gamma^{*}\right)}{4C_{i}+8\Gamma^{*}},$$

$$W_{p}=3\;\mathrm{TPU}+\frac{0.5\times V_{o}\times O}{C_{i}\times r}.$$

Here, A is the photosynthesis rate (equivalent to P_n_); W_c_, W_j_, and W_p_ are the modelled photosynthesis rate components permitted by Rubisco activity, RuBP regeneration, and triose phosphate utilization, respectively, before subtraction of R_d_; R_d_ is nonphotorespiratory CO_2_ release in the light; V_cmax_ is the maximum Rubisco carboxylation rate; C_i_ is the intercellular CO_2_ concentration; Γ^*^ is the photorespiratory CO_2_ compensation point in the absence of R_d_; K_c_ and K_o_ are the Michaelis constants of Rubisco for CO_2_ and O_2_, respectively; O is the O_2_ concentration; J is the electron transport rate supporting RuBP regeneration at the measurement irradiance; TPU is the triose phosphate utilization rate; V_o_ is the Rubisco oxygenation rate; and r is the dimensionless Rubisco specificity factor for CO_2_.

C_i_, Γ^*^, and K_c_ must use the same concentration units, and O and K_o_ must use the same units. In the W_p_ expression, O and C_i_ must also use the same concentration basis and units. A, W_c_, W_j_, W_p_, R_d_, and V_cmax_ are expressed in µmol CO_2_ m^−2^ s^−1^; J is expressed in µmol electrons m^−2^ s^−1^; and TPU is expressed in µmol triose phosphate m^−2^ s^−1^. V_o_ is expressed in µmol O_2_ m^−2^ s^−1^.

The maximum Rubisco carboxylation rate (V_cmax_), electron transport parameter supporting RuBP regeneration (J_max_), triose phosphate utilization rate (TPU), and carboxylation efficiency (CE) were calculated for each biological replicate from the CO_2_-response curves using Photosyn Assistant 1.1.2 and the equations above. The calculations used gas-exchange measurements at different CO_2_ concentrations and a constant PPFD of 250 µmol m^−2^ s^−1^, the same irradiance used for the other gas-exchange measurements. Jmax is reported as the electron transport parameter estimated from these curves under the measurement conditions. CE denotes the initial slope of the A/C_i_ response curve. Because the calculations were based on C_i_ rather than on the chloroplast CO_2_ concentration, these parameters were treated as estimates that may incorporate the effect of mesophyll conductance. The means and standard errors are presented in Table 1.

### 4.3. Photosystem II Photochemistry

Fluorescence induction curves were measured with an IMAGING-PAM fluorometer (Walz, Germany, Effeltrich). The plants were dark-adapted for 30 min before measurement. The leaves were then exposed to blue actinic light (450 nm; 250 µmol photons m^−2^ s^−1^) for 10 min, with superimposed saturating pulses. The measurement light was 0.5 µmol m^−2^ s^−1^. Saturating blue-light pulses (450 nm) were applied at an intensity of 5000 µmol m^−2^ s^−1^ for 800 ms. Calculations were performed using ImagingWin v.2.41a (Walz, Germany). The results are expressed in relative units. The effective quantum yields of photosystem II (Y(II)) and regulated (Y(NPQ)) and unregulated (Y(NO)) nonphotochemical energy dissipation were determined. Parameters were calculated using the standard equations for PAM fluorometry: Y(II) = (F′m − Fs)/F′m; Y(NPQ) = 1 − Y(II) − Y(NO); Y(NO) = Fs/Fm [37,38,39]. The same measuring spectrum and timing were used for every growth treatment. Photochemical acclimation developed throughout the 14-day spectral treatment; the blue actinic light was an integral part of the fluorescence protocol and was applied identically to all treatments.

### 4.4. Photosynthetic Pigments and Chloroplast Ultrastructure

#### 4.4.1. Photosynthetic Pigments

Photosynthetic pigments were extracted with 80% acetone in the dark and determined spectrophotometrically according to Lichtenthaler and Wellburn [40]. Absorbance was measured at 470, 646, and 663 nm. Pigment contents were expressed on a leaf dry-weight basis. Four independent biological replicates were analysed for each genotype-by-light-treatment combination (*n* = 4).

#### 4.4.2. Transmission Electron Microscopy and Chloroplast Morphology

Leaf fragments measuring 2–3 mm were fixed in 2.5% glutaraldehyde for 3 h and then incubated in 1% OsO_4_ for 24 h at 4 °C. Both solutions were prepared in 0.05 M cacodylate buffer (pH 7.2). The material was sequentially dehydrated twice for 15 min in 30% and 40% ethanol; twice for 30 min in 50% and 60% ethanol; overnight at 4 °C in 70% ethanol; for 1 h in 96% ethanol; and for 1 h in a 1:1 mixture of absolute ethanol and acetone. The samples were infiltrated with epoxy embedding medium (Fluka, cat. no. 2920114) in 1:8, 1:4, and 1:1 mixtures with acetone and then polymerized. Ultrathin sections were prepared using a Reichert U3 ultramicrotome, stained with 1% uranyl acetate, and examined with a LIBRA 120 electron microscope (Carl Zeiss, Germany, Oberkochen). The starch-grain area, grana area, stroma area, and plastoglobule number were quantified under all four light treatments (Figure 5A–D). Areas are expressed in µm^2^, and plastoglobule number is expressed per chloroplast. TEM analyses used four independent biological replicates for each genotype-by-light-treatment combination (*n* = 4). Five ultrathin sections and 20 micrographs were analysed for each experimental point. Sections and micrographs were subsamples and were not counted as independent biological replicates.

### 4.5. Relative Transcript Levels

Total RNA was isolated using the TRIzol method (Sigma-Aldrich, Burlington, MA, USA). RNA concentration and purity were assessed using a NanoDrop 2000 spectrophotometer (Thermo Fisher Scientific, Waltham, MA, USA). cDNA was synthesized using an M-MLV Reverse Transcriptase Kit (Fermentas, Waltham, MA, USA) and an oligo(dT)21 primer for the nuclear-encoded genes analysed in this study. RT–qPCR was performed using a CFX96 Touch Real-Time PCR Detection System (Bio-Rad, Hercules, CA, USA). Relative transcript levels (RQ) were calculated automatically by the instrument software, with normalization to *Actin1* (*ACT1* FJ532351.1) and *α-tubulin* (*TUBα*; NM_001347592.1) as reference genes. Four independent biological replicates were analysed for each genotype-by-light-treatment combination (*n* = 4), with at least three technical reactions per biological replicate.

Four functional groups of genes were analysed. Light signalling regulators included *PIF4* (phytochrome interacting factor 4; Solyc07g043580), *HY5* (elongated hypocotyl 5; Solyc08g061130), *COP1* (constitutive photomorphogenic 1; Solyc12g005950), and *SPA1* (suppressor of PHYA-105 1; Solyc10g011690). The phenylpropanoid-biosynthesis group included *FLS* (flavonol synthase; Solyc11g013110), *PAL* (phenylalanine ammonia-lyase), *CHS* (chalcone synthase), and *MYB12* (R2R3-MYB transcription factor 12; Solyc01g079620). Photosynthesis- and photoprotection-related genes included *PSBS* (photosystem II subunit S; Solyc06g060340), *ZEP* (zeaxanthin epoxidase; Solyc02g090890), VDE (violaxanthin de-epoxidase; Solyc04g050930), *RBCS* (ribulose-1,5-bisphosphate carboxylase/oxygenase small subunit), and *LHCB2* (light-harvesting chlorophyll a/b-binding protein 2). Antioxidant defence and guard-cell ion transport were characterized using *APX1* (cytosolic ascorbate peroxidase 1; Solyc09g007270), *SLAC1* (slow anion channel-associated 1), and *AHA2* (plasma-membrane H^+^-ATPase 2). The primer sequences are provided in Table S1. RQ values for each biological replicate were log_2_-transformed.

Means and SE were calculated across four independent biological replicates (*n* = 4) for each genotype-by-light-treatment combination. Table S2 presents log_2_(RQ) values. Within each light treatment, different lowercase letters indicate significant differences between WT and *cry1* according to one-way ANOVA followed by Duncan’s multiple range test (*p* < 0.05).

### 4.6. Phenolic Compounds and Antiradical Activity

UV-absorbing compounds were extracted with 1% hydrochloric acid in methanol, and the chlorophyll was removed with chloroform. The absorbance of the aqueous methanolic phase was measured at 327 nm and normalized to the leaf fresh weight. Four independent biological replicates were analysed for each genotype-by-light-treatment combination (*n* = 4), with three technical measurements per biological replicate.

The total phenolic content was determined using Folin–Ciocalteu reagent. The reaction mixture contained 0.05 mL of extract and 1.95 mL of reagents; the extract background absorbance was subtracted from the measured absorbance. The results are expressed as milligrams of gallic acid equivalents per gram of fresh weight.

The total flavonoid content was determined colorimetrically after the sequential addition of NaNO_2_, AlCl_3_, and NaOH and calculated from the (+)-catechin calibration relationship. The results are expressed in arbitrary units.

The ability of the extracts to scavenge the ABTS radical cation was determined from its discolouration after the addition of 0.02 mL of extract to 2.00 mL of reaction medium. The activity in the DPPH radical assay was determined from the decrease in the optical signal after the addition of 0.005–0.020 mL of extract to 2.00 mL of reaction medium, depending on the activity range. For both methods, ΔA = A0 − At was calculated, and the Trolox calibration relationship ΔA = Kc + B was used. Electron-transfer antioxidant assays were performed according to the methods of Apak et al. [41].

The results are expressed as milligrams of Trolox equivalents per gram of fresh weight. Three technical replicates were analysed in each of the four biological replicates for every genotype-by-light-treatment combination (*n* = 4).

### 4.7. Statistical Analysis

Physiological, biochemical, molecular, and TEM analyses were performed using four independent biological replicates for each genotype × light treatment combination (*n* = 4). Each biological replicate consisted of one individual plant. Fully expanded leaves from the second and third nodes were sampled for analysis. RT–qPCR included at least three technical replicates per biological replicate. Technical reactions, sections, and micrographs were not treated as independent biological replicates. Data are expressed as means ± standard error of the mean (SE), calculated across the four biological replicates. Before analysis of variance, normality was assessed using the Shapiro–Wilk test and homogeneity of variance using Levene’s test. Differences between genotypes within each light treatment were analysed using one-way analysis of variance (ANOVA) followed by Duncan’s multiple range test. Statistical analyses were performed in SigmaPlot 12.3 (Systat Software, San Jose, CA, USA). Differences were considered statistically significant at *p* < 0.05. Different letters in the figures and tables indicate significant differences between genotypes within the same light treatment.

## 5. Conclusions

The *cry1* line differed from WT in photosynthesis rate, stomatal conductance, photosystem II energy partitioning, pigment accumulation, chloroplast ultrastructure, and transcript levels across the tested light treatments. Under BL, increased chlorophyll content and grana area in *cry1* were accompanied by a lower photosynthesis rate and lower Y(II). The greatest relative reduction in photosynthesis rate occurred under BL/GL: the photosynthesis rate and stomatal conductance were approximately 35% lower, and Vcmax, Jmax, and TPU were 48–57% lower in *cry1* than in WT. Under BL/GL, lower Y(II) was accompanied by higher Y(NO), and the grana area was lower in *cry1*. The starch-grain area, stroma area, and plastoglobule number showed small differences that were not statistically significant. These results support a contribution of CRY1a to acclimation to light quality. We propose that spectral modulation of CRY1-dependent responses affects the balance between CO_2_ supply and carbon utilization, promoting photoprotective feedback when carbon metabolism is restricted.

## Figures and Tables

**Table 1:** Parameters calculated from A/Ci curves in WT and *cry1* mutant plants.

Parameter	Genotype	WFL	BL	BL/GL	GL
Maximum Rubisco carboxylation rate, Vcmax, µmol m^−2^ s^−1^	WT	12.7 ± 1.9 ^a^	13.8 ± 1.3 ^a^	12.2 ± 1.8 ^a^	9.3 ± 0.8 ^a^
	*cry1*	9.7 ± 1.8 ^a^	12.0 ± 1.3 ^a^	6.4 ± 1.5 ^b^	7.6 ± 1.2 ^a^
Electron transport parameter, Jmax, µmol m^−2^ s^−1^	WT	41.5 ± 4.2 ^a^	42.8 ± 5.6 ^a^	39.5 ± 3.2 ^a^	21.0 ± 0.4 ^a^
	*cry1*	29.7 ± 3.4 ^a^	38.5 ± 4.1 ^a^	19.0 ± 2.8 ^b^	17.5 ± 2.5 ^a^
Triose phosphate utilization rate, TPU, µmol m^−2^ s^−1^	WT	3.3 ± 0.8 ^a^	3.4 ± 0.5 ^a^	3.0 ± 0.5 ^a^	1.5 ± 0.2 ^a^
	*cry1*	2.3 ± 0.7 ^a^	3.0 ± 0.3 ^a^	1.3 ± 0.3 ^b^	1.2 ± 0.2 ^a^
Carboxylation efficiency (CE), µmol CO_2_ m^−2^ s^−1^ Pa^−1^	WT	0.074 ± 0.022 ^a^	0.096 ± 0.021 ^a^	0.085 ± 0.012 ^a^	0.062 ± 0.018 ^a^
	*cry1*	0.049 ± 0.012 ^a^	0.086 ± 0.019 ^a^	0.031 ± 0.007 ^b^	0.052 ± 0.016 ^a^

Values are means ± SE of four independent biological replicates (*n* = 4). Vcmax, maximum Rubisco carboxylation rate; Jmax, electron transport parameter supporting RuBP regeneration estimated from the CO_2_-response curves at 250 µmol m^−2^ s^−1^; TPU, triose phosphate utilization rate; CE, carboxylation efficiency. Within each light treatment, different letters indicate significant differences between genotypes according to one-way ANOVA followed by Duncan’s multiple range test (*p* < 0.05).

## Data Availability

The data presented in this study are available from the corresponding author upon reasonable request.

## Supplementary Materials

The following supporting information can be downloaded at https://www.mdpi.com/article/10.3390/plants15213297/s1, The supplementary tables are included at the end of this document: Table S1: Sequences of primers used for qRT-PCR; Table S2: Relative transcript levels in WT and cry1 plants under WFL, BL, BL/GL, and GL.

## Abbreviations

BL, blue light; CE, carboxylation efficiency; CRY1a, cryptochrome 1a; E, transpiration rate; GL, green light; gs, stomatal conductance; Jmax, electron transport parameter supporting RuBP regeneration estimated from CO_2_-response curves; Pn, photosynthesis rate; RQ, normalized relative transcript level; RuBP, ribulose-1,5-bisphosphate; TPU, triose phosphate utilization; Vcmax, maximum Rubisco carboxylation rate; WFL, white fluorescent light; WT, wild type; Y(II), effective quantum yield of photosystem II; Y(NPQ), quantum yield of regulated nonphotochemical energy dissipation; Y(NO), quantum yield of unregulated nonphotochemical energy dissipation.
