# Supplementary material for: Effects of Blue and Green Light on Pigment Content, Photosynthesis, and Chloroplast Structure in CRY1a-Deficient Tomato Mutants
Source: Plants (Basel). 2026 Oct 26;15(21):3297. doi: 10.3390/plants15213297 (PMC13765924; doi:10.3390/plants15213297)
Supplement: Supplementary file 1 [file plants-15-03297-s001.zip › plants-4626358-supplementary.pdf]

plants

MDPI

Supplementary Materials Table S1. Sequences of primers used for qRT-PCR.

Gene	Forward primer (5′-3′)	Reverse primer (5′-3′)
PIF4	GAGTTGTTGTGGCGAAACGG	TAGCCACGCGACAGTTTCAT
HY5	AAATGCAAGAGCAAGCGACG	CGGCCGATACTCCATCTCTTC
COP1	TGAACCTGCAGATGCACACT	AATCAACACTCCAAGCCCGT
SPA1	AGAACACTCAGCCCTGCTTC	GGTACCGAGGTTGAATGCCA
FLS	CGACAGAAGCTGGGATTTCCT	GGATCCATTCGCCGTCCTTT
PAL	TGCAGCTCCAACTACCCTTT	ATTCGTCCTCGAAAGCTCCA
CHS	CCGTGAACCCAGTGAATCTC	CTCACGTAGGTGTCCGTCAAT
MYB12	AGCTTGTGATAGTGCCATGG	ATCACCACGTCTGGCATAATC
PSBS	TGTTGGCCGTGTTGCTATGA	TTGCCAGGAGGGATAACAGC
ZEP	CCTTGTAGGAGCTTGGAAAATGGG	TCTTATGCCATCAGCACCAACCAG
VDE	TGTCAAGTTGGATCAAGGCT	CATCTCAAACCCTGCATGTG
RBCS	GTTGCCTATGTTTGGGTGC	TGCTTGTGGGTATGCCTTT
LHCB2	GAGAAGACCGTCCTAAGT	TAAAGCACCAAGCATAGC
APX1	ATACCTTGGGAAGGGCACAC	TGACGGCAACTGTAAGAGCC
SLAC1	ACATAACTCAGGAGACAAAGAGTGC	ATAGTTCGGACAGTTAGTCTCGATTA
AHA2	GCTTTGATTTTCGTGACCCGT	TGGCAACCAATTGGGCAATCA
Actin1	TTAGCAACTGGGATGACATGGA	CCTGAATGGCAACATACATAGCA
TUBα	ACAACTTTGCCCGTGGACAT	TGCTCAAGAAGGGAGTGGGT

Table S2. Relative transcript levels in WT and *cry1* plants under WFL, BL, BL/GL, and GL.

**Gene**	**WFL WT**	**WFL *cry1***	**BL WT**	**BL *cry1***	**BL/GL WT**	**BL/GL *cry1***	**GL WT**	**GL *cry1***
PIF4	1.64 ± 0.14^{b}	2.29 ± 0.11^{a}	1.86 ± 0.12^{a}	-0.18 ± 0.10^{b}	1.12 ± 0.03^{a}	1.28 ± 0.08^{a}	0.70 ± 0.10^{a}	0.38 ± 0.09^{b}
HY5	-0.47 ± 0.08^{b}	0.41 ± 0.06^{a}	3.33 ± 0.09^{a}	-2.56 ± 0.13^{b}	1.79 ± 0.07^{a}	0.00 ± 0.04^{b}	-1.36 ± 0.20^{a}	-2.18 ± 0.16^{b}
COP1	0.29 ± 0.11^{b}	1.98 ± 0.11^{a}	1.72 ± 0.13^{a}	-0.34 ± 0.07^{b}	1.63 ± 0.04^{a}	0.98 ± 0.06^{b}	-0.07 ± 0.07^{a}	-0.74 ± 0.06^{b}
SPA1	-0.76 ± 0.06^{b}	0.01 ± 0.12^{a}	1.11 ± 0.11^{a}	0.00 ± 0.05^{b}	-1.94 ± 0.07^{b}	0.43 ± 0.08^{a}	-1.32 ± 0.09^{b}	-1.03 ± 0.07^{a}
FLS	-1.09 ± 0.04^{a}	-1.43 ± 0.05^{b}	3.10 ± 0.02^{a}	-0.86 ± 0.04^{b}	1.97 ± 0.08^{a}	1.40 ± 0.08^{b}	-0.22 ± 0.08^{a}	-1.22 ± 0.12^{b}
PAL	-1.15 ± 0.10^{b}	0.54 ± 0.06^{a}	0.55 ± 0.05^{a}	-3.06 ± 0.07^{b}	2.02 ± 0.14^{a}	0.93 ± 0.05^{b}	-1.69 ± 0.11^{a}	-3.64 ± 0.09^{b}
CHS	-1.89 ± 0.15^{a}	-5.06 ± 0.08^{b}	3.10 ± 0.09^{a}	-3.47 ± 0.13^{b}	0.52 ± 0.12^{a}	0.38 ± 0.09^{a}	0.25 ± 0.04^{a}	-1.00 ± 0.07^{b}
MYB12	0.57 ± 0.03^{a}	-2.32 ± 0.05^{b}	1.09 ± 0.02^{a}	-0.51 ± 0.10^{b}	0.00 ± 0.09^{b}	1.04 ± 0.10^{a}	0.12 ± 0.09^{a}	-1.03 ± 0.05^{b}
PSBS	-0.67 ± 0.08^{a}	-1.69 ± 0.12^{b}	1.77 ± 0.07^{a}	0.00 ± 0.11^{b}	-1.94 ± 0.06^{b}	1.34 ± 0.16^{a}	-2.00 ± 0.13^{a}	-1.84 ± 0.04^{a}
ZEP	-1.84 ± 0.17^{a}	-2.25 ± 0.10^{b}	2.41 ± 0.03^{a}	0.10 ± 0.06^{b}	0.23 ± 0.08^{a}	-0.22 ± 0.09^{b}	-0.15 ± 0.15^{a}	-0.62 ± 0.07^{b}
VDE	-4.64 ± 0.06^{a}	-6.64 ± 0.09^{b}	-5.06 ± 0.05^{a}	-6.64 ± 0.09^{b}	1.72 ± 0.09^{b}	2.10 ± 0.13^{a}	-2.94 ± 0.08^{a}	-6.64 ± 0.07^{b}
RBCS	-0.56 ± 0.07^{b}	-0.81 ± 0.13^{a}	1.58 ± 0.05^{a}	0.10 ± 0.07^{b}	-3.84 ± 0.08^{b}	0.64 ± 0.04^{a}	-1.51 ± 0.05^{a}	-1.64 ± 0.08^{a}
LHCB2	-2.64 ± 0.09^{a}	-4.64 ± 0.12^{b}	-0.42 ± 0.18^{b}	0.10 ± 0.11^{a}	-6.64 ± 0.04^{b}	-1.00 ± 0.04^{a}	-1.25 ± 0.07^{b}	-0.76 ± 0.07^{a}
APX1	-0.89 ± 0.12^{a}	-1.94 ± 0.03^{b}	-0.04 ± 0.05^{a}	-1.09 ± 0.05^{b}	0.00 ± 0.05^{a}	0.15 ± 0.10^{a}	-0.10 ± 0.05^{a}	-0.27 ± 0.16^{a}
SLAC1	0.00 ± 0.10^{b}	1.70 ± 0.12^{a}	1.59 ± 0.06^{a}	-0.51 ± 0.09^{b}	-0.49 ± 0.06^{b}	1.01 ± 0.09^{a}	0.44 ± 0.05^{a}	0.25 ± 0.14^{a}
AHA2	-1.18 ± 0.08^{b}	1.04 ± 0.14^{a}	0.78 ± 0.16^{a}	-1.94 ± 0.09^{b}	1.72 ± 0.15^{a}	1.21 ± 0.07^{b}	-1.09 ± 0.04^{a}	-1.60 ± 0.05^{b}

Values are means ± SE of $\log_2$(RQ) calculated across four independent biological replicates (n = 4 plants; one plant per biological replicate), each with at least three technical reactions. RQ values were calculated automatically by the instrument software, with normalization to *Actin1* and α-tubulin (*TUBα*) as reference genes, and were then $\log_2$-transformed. Within each light treatment, different letters indicate significant differences between genotypes according to one-way ANOVA followed by Duncan's multiple range test ($p < 0.05$).
